# AAPM Medical Physics Practice Guideline 5.a.: Commissioning and QA of Treatment Planning Dose Calculations — Megavoltage Photon and Electron Beams

**DOI:** 10.1120/jacmp.v16i5.5768

**Published:** 2015-09-08

**Authors:** Jennifer B. Smilowitz, Indra J. Das, Vladimir Feygelman, Benedick A. Fraass, Stephen F. Kry, Ingrid R. Marshall, Dimitris N. Mihailidis, Zoubir Ouhib, Timothy Ritter, Michael G. Snyder, Lynne Fairobent

## Abstract

The American Association of Physicists in Medicine (AAPM) is a nonprofit professional society whose primary purposes are to advance the science, education and professional practice of medical physics. The AAPM has more than 8,000 members and is the principal organization of medical physicists in the United States.

The AAPM will periodically define new practice guidelines for medical physics practice to help advance the science of medical physics and to improve the quality of service to patients throughout the United States. Existing medical physics practice guidelines will be reviewed for the purpose of revision or renewal, as appropriate, on their fifth anniversary or sooner.

Each medical physics practice guideline represents a policy statement by the AAPM, has undergone a thorough consensus process in which it has been subjected to extensive review, and requires the approval of the Professional Council. The medical physics practice guidelines recognize that the safe and effective use of diagnostic and therapeutic radiology requires specific training, skills, and techniques, as described in each document. Reproduction or modification of the published practice guidelines and technical standards by those entities not providing these services is not authorized.

The following terms are used in the AAPM practice guidelines:

- Must and Must Not: Used to indicate that adherence to the recommendation is considered necessary to conform to this practice guideline.
- Should and Should Not: Used to indicate a prudent practice to which exceptions may occasionally be made in appropriate circumstances.

## I. INTRODUCTION

The treatment planning system (TPS) is an essential component of external beam radiation therapy. TPSs are used to plan the beam arrangements, energies, field sizes, fluence patterns, and modifiers that provide optimum dose distributions to treat disease and minimize dose to the healthy tissues. The accuracy of the dose calculations is paramount for safe and efficacious treatment delivery. A substantial (but not exclusive) part of commissioning a TPS is ensuring that the radiation beam parameters, and other data affecting the accuracy of the dose calculation, are adequately modeled in the system and are properly validated. These tasks are the subject of this Medical Physics Practice Guideline (MPPG).

There are a variety of radiation oncology TPSs, from widely used commercial systems to special purpose systems, with limited application to a specific delivery modality. The dose calculation algorithms range from simple correction‐based algorithms to complex Monte Carlo calculations. For minimum tolerance values and evaluation criteria, this report assumes use of model‐based photon dose algorithms with 3D heterogeneity corrections including convolution/superposition with point kernels, grid‐based Boltzmann transport equation solvers, and Monte Carlo algorithms.^1^, ^2^, ^3^ For electron beams, pencil beam or Monte Carlo dose algorithms are assumed. However, the commissioning process and validation tests should be applied to all external photon and electron beam algorithms in clinical practice at a given facility.

A given TPS may include multiple dose calculation algorithms. Prior to beginning the commissioning, a Qualified Medical Physicist (QMP)^*^ must select a dose computation algorithm(s) for commissioning. The QMP must have a clear understanding of the algorithm(s) chosen. The QMP must understand how the commissioning measurements relate to the model parameters and how each one affects the resulting dose distributions.

The QMP should generate a reasonable estimate of the time required to acquire data, and model and verify the dose algorithms. Assuming 12–16 QMP work hours per day (1.5 to 2.0 FTEs), reasonable time estimates are two to four weeks for a single energy photon beam and six to eight weeks for two photon energies and five electron energies.^4^, ^5^ This will depend strongly on how much commissioning data need to be collected and the availability and experience of the QMP(s) involved, the adequacy and availability of the equipment used, and the access to the accelerator. Addition of a second algorithm for a given beam will increase commissioning time and effort. The recommendations in this report are based on the minimum requirements for well‐established commercial systems with available manufacturer's guidance on the commissioning process. The goals and scope of this document are defined below.

### A. Goals

A QMP is responsible for the commissioning and quality assurance (QA) of TPSs in a clinical radiation therapy department. This document is part of a series of MPPG commissioned by the American Association of Physicists in Medicine (AAPM) and intended to succinctly state the minimum acceptable standards for various aspects of clinical medical physics.

**Figure 1:**
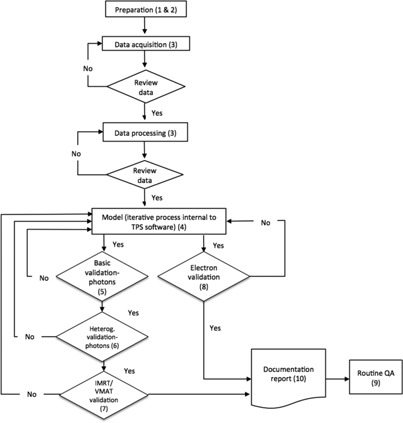
Workflow of TPS dose algorithm commissioning, validation, and routine QA. The numbers refer to sections of this report.

Many guidelines, task group reports, and other peer reviewed journal articles have been published on the topic of TPS commissioning, evaluation and QA.^4^, ^5^, ^6^, ^7^, ^8^, ^9^ TPS vendors may provide detailed manuals for their systems. While the implementation of robust and comprehensive QA programs recommended in other AAPM reports is strongly encouraged, the overall objective of this MPPG is to provide an overview of the minimum requirements for TPS dose algorithm commissioning and QA in a clinical setting. In this report the term “commissioning” includes beam data acquisition, modeling, and verification. The routine QA and validation tests required following a software or hardware update affecting the dose algorithm are subsets of this work and are, therefore, also covered by this report. Figure 1 depicts activities that are part of commissioning. The specific goals for this report are to:

- Clearly identify and reference applicable portions of existing AAPM reports and peer‐reviewed articles for established commissioning components.
- Provide updated guidelines on technologies that have emerged since the publication of previous reports.
- Provide guidance on validation tests for dose accuracy and constancy (select downloadable datasets/contours and beam parameters are provided for optional use).
- Provide guidance on tolerance values and evaluation criteria for clinical implementation.
- Provide a checklist for commissioning processes and associated documentation.

### B. Tolerances values and evaluation criteria

Modeling the commissioning data in the TPS is an iterative process that includes compromises in accuracy over the range of clinical scenarios that could be encountered. Consequently, some aspects of the validation tests may show excellent agreement, while others may show poorer agreement. Accurate model verification is affected by both measurement and model limitations. Some components of the dose distribution may be difficult to measure accurately (e.g., detector overresponding to low‐energy photons in the low‐dose tail profiles) while, in other circumstances, the treatment planning system may not model the dose well even when appropriate and accurate input data are used.^10^, ^11^ The desired accuracy should be driven by the needs of the clinic. The tolerance values and evaluation criteria in this MPPG represent a compromise between a number of factors:

1. Avoiding values that are too “tight” and may be unreasonable or unachievable over the investigated range of field sizes, depths, off‐axis positions, test setups, and beam modifiers.
2. Avoiding values that are too “loose” and could, therefore, result in approval of a sub‐optimal model.
3. The need for a simple, generic set of evaluation criteria, as opposed to a complex matrix of test scenarios and tolerances for different parts of the model which could potentially lead to confusion.

Each validation section (basic photon, heterogeneity, IMRT/VMAT and electrons) has its own criteria. Although the basic photons are often modeled (and verified) first, it is important to note that, if the model just meets the basic photon tolerance values, it will likely provide unacceptable results when IMRT/VMAT evaluation criteria are considered. Parameter values will likely need to be adjusted for IMRT/VMAT modeling but not to the extent that it would change the passing of the basic validation tests; nonetheless, all changes must be validated.

The tolerance values for the basic photon tests are widely accepted for static photon beams under conditions of charged particle equilibrium. The tolerances for the simple heterogeneity and basic electron beam validation tests are considered widely accepted and, therefore, are stated as minimum tolerances, as well. However, given that there are no widely accepted minimum tolerance values for the other verification tests in this MPPG, (including those for VMAT/IMRT), those evaluation criteria must not be interpreted as mandatory or regulatory tolerances. Rather, they are values defined as points for further investigation, possible improvement, and resolution. All the tolerances and criteria in this report are based on a combination of published guidelines, the dosimetric audits performed by the Imaging and Radiation Oncology Core — Houston (IROC Houston; formerly the Radiological Physics Center, RPC^†^), and the experience of the authors. Users are encouraged to not only meet these tolerances, but also strive to achieve dosimetric agreement comparable to that reported in the literature for their particular algorithm(s).

### C. Scope

The scope of this report is limited to the commissioning and QA of the beam modeling and calculation portion of a TPS where:

- External photon and electron treatment beams are delivered at typical source‐to‐surface distance (SSDs) using a gantry‐mounted radiation source including conventional and small fields used in IMRT, VMAT, and helical tomotherapy delivery.
- Modern dose algorithms are utilized, including corrections for tissue heterogeneity.
- The multileaf collimator (MLC) is used as the primary method of shaping the beam aperture or modulating the fluence for treatments.
  - Areas of treatment planning commissioning and QA that fall *outside* the scope of this report include noncommercial planning systems, small static shaped fields less than $2\times 2\;{\text{cm}}^{2}$ such as those used in stereotactic radiosurgery (SRS), secondary monitor unit validation and other such ancillary software, optimization and leaf sequencing algorithms, methods involving biological modeling (including tumor control and normal tissue complication probability), and all nondosimetric components of the planning system. Nondosimetric components include (but are not limited to) dataset management and presentation, coordinate systems, image generation, image registration, anatomical structures, and functions dependent on anatomy (e.g., dose‐volume histograms, beam's eye view displays).

### D. Intended users and precautions

The intended user of this document is the QMP. Hospital and clinic administration are also encouraged to use this report as a reference for an explanation of process, time, and resource requirements.

This document does not contain specifics on the use of commercially available TPS software. The QMP must be properly trained in the use of the planning software and related systems prior to clinical implementation. In addition, the configured treatment machine type and the planning system should have a history of compatibility and agreement between calculated and delivered dose.

### E. Acronyms/abbreviations

- AAPM – American Association of Physicists in Medicine
- C/S – Convolution/Superposition
- CC – Collapsed Cone
- CT – Computed Tomography
- D – Dose
- DTA – Distance to Agreement
- GBBS – Grid‐Based Boltzmann Transport Equation Solver
- IGRT – Image‐Guided Radiation Therapy
- IMRT – Intensity‐Modulated Radiation Therapy
- linac – Linear Accelerator
- MC – Monte Carlo
- MLC – MultiLeaf Collimator
- MPPG – Medical Physics Practice Guidelines
- MU – Monitor Unit
- OAR – Organ at Risk
- PB – Pencil Beam
- PDD – Percent Depth Dose
- QA – Quality Assurance
- QMP – Qualified Medical Physicist
- SRS – Stereotactic Radiosurgery
- SSD – Source‐to‐Surface Distance
- TLD – Thermoluminescent Dosimeter
- TPS – Treatment Planning System
- VMAT – Volumetric‐Modulated Arc Therapy

## 2. STAFF QUALIFICATIONS AND RESPONSIBILITIES

Medical Physicist, the QMP as defined in AAPM Professional Policy 1, must be competent to practice independently in the subfield of Therapeutic Medical Physics.

## 3. DATA ACQUISITION AND PROCESSING

This section describes the methods of acquiring the data necessary for modeling a treatment beam. The linac configuration and performance should be tuned and accepted prior to taking any measurements for commissioning. The QMP should understand the details of the required modeling data and should follow the recommendations from the TPS vendor for the required dataset. The authors of this report strongly discourage reducing the required dataset because of time or convenience. The vendor may be a valuable resource and should be consulted in such a scenario. If the TPS is being commissioned in parallel with the commissioning of a new linear accelerator, then a full set of new modeling data is required. If a new TPS and/or new algorithm are being commissioned on an existing linear accelerator, then existing data could be used, provided they are verified (compared with recent QA measurements to assess any changes in beam characteristics) and meet vendor requirements. However, additional data may also be required. It may be useful to acquire data that will be used for verification (refer to Sections 6–9 of this report) at the same time commissioning data are collected.

### A. Equipment

The QMP should identify the required equipment in advance. The QMP must verify the functionality, correct operation, and calibration status (if applicable) of equipment and understand the limitations and uncertainties of each device in regards to the intended measurement. Table 1 summarizes some of the detectors appropriate for use to obtain the data under various conditions. Not all detectors are necessary, provided that an appropriate detector is identified for each task. Table 2 lists the minimum required additional equipment for a typical commissioning effort.

The QMP must be aware of setup variables and measurement uncertainties associated with beam data collection. Choices such as scanning speed, detector size, noise, data processing, detector orientation, and a myriad of other factors, can significantly alter the measured results. Task Group (TG) 106^(5)^ provides an excellent summary of these topics and should be referenced for additional details. The achievable level of accuracy should also be considered prior to beginning the commissioning process, as this will affect equipment choices and measurements.

**Table 1:** Detectors suitable for TPS commissioning and validation of photon and electron beams

*Detector*	*Use*	*Comments*	*Reference*
Scanning ion chambers	Beam scanning for photons and electrons	Typical scanning chambers have an air cavity of 4−6 mm diameter, (minimum of 2 chambers for measurement and reference)	TG‐106 (Das et al.^(5)^)
Electron diodes and film	Beam scanning for electrons, output factors (film)	QMP must confirm the effective point of measurement	TG‐25 (Khan et al.^(45)^), TG‐70 (Gerbi et al.^(46)^)
Small field detectors	• Small field scanning & output factors^a^,	Carefully select the detector type and size to fit the application.	TG‐106 (Das et al.^(5)^), TG‐120 (Low et al.^(18)^) Yunice, et al.^(16)^
	• IMRT/VMAT point measurement	When scanning for penumbra, diodes are recommended.	
	• MLC intraleaf measurement & penumbra		
Large ion chamber	Aggregate MLC transmission factors	Interleaf transmission	LoSasso et al.^(20)^
Film and/or array detector	2D dose distributions, including dynamic/virtual wedge and planar fluence maps, intraleaf measurements^b^	• Absolute dosimetry preferred; relative dosimetry adequate.	TG‐106 (Das et al.^(5)^), TG‐120 (Low et al.^(18)^), IAEA TRS‐430^(7)^
		• Desirable if the device can be mounted on the gantry and/or in a phantom at different geometries	

a If a diode detector is used for small field measurements, a “daisy chain” approach is recommended to minimize the energy dependence effects; the diode is first cross‐compared with an ion chamber for a $4\times 4\;{\text{cm}}^{2}$ field and then is used to measure the smaller fields.

b Using film for intraleaf transmission is usually less precise than interleaf transmission.

**Table 2:** Equipment required for TPS commissioning of photon and electron beams

*Equipment*	*Use*	*Comments*	*Reference*
3D water phantom	Beam scanning	Must have sufficient scanning range and lateral/depth scatter	TG‐106 (Das et al.^(5)^), TG‐70 (Gerbi et al.^(46)^)
Electrometers and cables	Beam scanning, output calibration, relative and absolute dosimetry	ADCL calibration, low noise and leakage with wide dynamic range and linear response	TG‐106 (Das et al.^(5)^)
Buildup cap or miniphantom	In‐air output factor measurement	Measurements required for some planning systems, most second check systems	Yunice, et al.^(16)^
Water‐equivalent phantom material in slab form	Buildup and backscatter for measurements	>20 cm of total thickness in varying increments, width and length ≥30 cm, cavity for detector(s)	TG‐106 (Das et al.^(5)^), TG‐120 (Low et al.^(18)^), IAEA TRS‐430^(7)^
CT density phantom	CT number to electron or mass density calibration	Should include tissue‐equivalent materials spanning the clinical range of low‐density lung to high‐density bone.	TG‐66 (Mutic et al.^(13)^)
Heterogeneity phantom with lung‐equivalent material	End‐to‐end testing	Include cavities for detectors, useful for annual QA reference test	TG‐65 (Papanikolaou & Stathakis^(26)^), IAEA TRS‐430^(7)^
Anthropomorphic phantom	Anatomic model testing, end‐to‐end testing, use testing	Include cavities for detectors	IAEA TRS‐430^(7)^
Software for data processing	Processing, comparing, and analyzing profiles, depth‐dose curves, and other beam data	May be included with the 3D water tank scanning software	TG‐106 (Das et al.^(5)^)
IMRT/VMAT or arc therapy phantom	VMAT or arc therapy	Options include a solid phantom holding a planar array, 3D detector arrays, film inside a phantom, other	TG‐120 (Low et al.^(18)^)

### B. Data acquisition for CT calibration

The dose calculation model will typically be commissioned based on dose measurements made in water and air. Dose calculation in heterogeneous media is dependent on the correct mapping of voxel intensities in a CT scan to some physical descriptor that can be used in the algorithm — typically physical or electron density (or less commonly, chemical composition^(12)^ — in the form of a “CT‐density” table.^(13)^

The QMP must consider the range of clinically relevant densities and scan parameters (kVp) as important components of the dose algorithm commissioning process. Materials used for CT number mapping must range from air ($∼0.001\;g/{\text{cm}}^{3}$) to high‐density material ($∼2\;g/{\text{cm}}^{3}$), including densities to mimic lung ($∼0.3\;g/{\text{cm}}^{3}$) and dense bone ($∼1.4-1.9\;g/{\text{cm}}^{3}$). High‐density calibration points (such as gold or titanium) may also be required. Image data should be evaluated over a large volume of each density plug to determine an average CT number for each density. A separate CT density curve should be developed and validated for the image guidance system if those CT datasets will be used for dose calculation. It is recommended that scanner‐specific calibration curves be obtained.

### C. Data acquisition for IMRT/VMAT delivery

The acquisition of data for conventional beam modeling is well documented.^(5)^ This report expands those recommendations to address the additional measurement considerations when modeling small beam apertures and MLC parameters characteristic of IMRT/VMAT delivery. The challenges of small field dosimetry have been well documented and require extremely careful measurement setup and use of an appropriate detector.^14^, ^15^, ^16^, ^17^ Low et al.^(18)^ provide an overview of IMRT dosimetry instruments and methods.

Dosimetry for small fields is often extrapolated by TPSs. MLCs also display considerable design variation between manufacturers.^(19)^ Therefore, measurements to verify both small fields and MLC characteristic are crucial to IMRT/VMAT dose calculation accuracy.

- Even if not specified by the TPS vendor, the QMP should measure percent depth dose (PDD) with a small‐volume detector down to a field size of $2\times 2\;{\text{cm}}^{2}$ or smaller for comparison with dose calculation.
- Vendor recommendations for measuring MLC intraleaf and interleaf transmission and leaf gap should be followed using a large detector if an average intra‐ and interleaf value is specified. For separate measurements, a small chamber should be used under the leaf and film should be used for interleaf leakage measurements.^(20)^
- Leaf‐end penumbra should be obtained with a small detector (such as a diode or micro‐chamber) to avoid volume‐averaging effects.
- Leaf timing for binary MLC systems should be verified using film or exit detector measurements.^(21)^
- Small field output factors (down to $2\times 2\;{\text{cm}}^{2}$ or smaller) should be measured for beam modeling and/or verification.^15^, ^22^

### D. Review of data

All data used in the modeling process must be reviewed both before and after entry into the planning system. There are three recommended components to this review.

Acquired data must be reviewed for potential setup and measurement errors prior to importing data into the TPS. Inverse square effects, beam divergence, expected beam energy changes with field size, and other well‐known characteristics should be validated (this is often easily performed by review of graphical display of the results). Crossbeam profiles at varying depths and field sizes can be superimposed on the same plot to identify trends. Depth‐dose plots can be analyzed in a similar fashion.

The data should be compared if possible to a reference dataset from the same type of, or a nearly identical, machine to identify systematic anomalies in either setup or machine properties. Points representing the middle, as well as extremes, of the data should be validated in this manner. Tolerance levels for this step cannot be provided since each machine is unique; however, the QMP will often find published papers or conference proceedings that describe typical beam properties and their expected variation.^4^, ^23^, ^24^, ^25^ MLC transmission factors should be compared to the published results obtained with the same MLC and energy.^(18)^

After the data are entered into the planning system, they must be re‐evaluated for potential processing errors (e.g., problems during import, smoothing, mirroring). A combination of graphical review and spot‐checking can be used.

## 4. MODEL WITHIN TPS SOFTWARE

Once the measured data and machine parameters are entered into the modeling module of the TPS, the actual beam modeling should be completed according to vendor instructions. For some TPSs, the linac vendor provides a predefined model or performs the modeling using customer supplied data; however, the validation process is still necessary and the vendor should provide reference data for comparison with QMP measurements.

Modeling is an iterative process, with parameters adjusted to optimally agree with the data used for comparison. The amount of adjustment available to the user varies between TPS vendors. Regardless of how much latitude exists in adjusting parameters, the QMP must understand how the measurements relate to the model parameters, and how each one (and its magnitude) will affect the resulting dose distribution. The QMP must understand the trade‐offs; the model is just that, a “model”, and will therefore not fit the measured data under all measurement conditions with the same accuracy. The QMP should evaluate the goodness of the fit based on qualitative assessment of the dose distribution (PDD and profiles) and use quantitative metrics within the modeling software.

After assessing the quality of the modeling within the TPS beam commissioning (or physics) application, this report recommends additional tests to validate the dose calculations (Sections 5–8). The results from each test should be used to adjust the model (or tune the machine in the case of matched or “twinned” systems), as needed. Sections 5–7 should be carried out in order, meaning that the basic validation testing in homogeneous media should be completed prior to testing in heterogeneous media.

The IMRT/VMAT dose is usually the last photon validation process. It is important to note that there are special considerations for modeling the MLC that strongly affect the algorithm's ability to correctly compute dose from an aggregate of small fields as used in IMRT/VMAT. Leaf transmission and/or dosimetric leaf gap offset can often be used to improve agreement between measured and calculated dose. Therefore, if changes are made to basic photon parameters in the iterative IMRT/VMAT modeling process, the basic photon validation must be reconfirmed.

## 5. PHOTON BEAMS: BASIC DOSE ALGORITHM VALIDATION

The basic photon beam dose validation tests described in this section must be completed for each configured beam. A “configured beam” is typically distinguished as a unique energy and/or accelerator head model configuration. A 6 MV, 6 MV SRS, and 6 MV flattening filter‐free (FFF) are all considered unique beams. Each physical wedge is a unique beam because of its independent energy fluence spectrum and therefore must be separately validated. Nonphysical wedges can be considered an extension of the corresponding open field, and only one additional validation test is presented in this report (Test 5.9 described below). However, additional validation of nonphysical wedges may require supplemental testing, depending on the configuration. The typical setup for the measurement of basic algorithm validation tests will be a static gantry angle pointing directly down with collimator rotated as needed to acquire the appropriate data point(s).

Much of the validation data can be acquired using a scanning water phantom; however, this task group considers an array detector appropriate for a subset of tests. Validation plans should be created by the clinical users of the system and should exploit typical clinical processes. While it is good practice to use field configurations for validation that were not used for modeling for the majority of the tests, it is efficient to collect the validation data at the same time as the modeling data are acquired.

### A. Validation tests

Table 3 summarizes three tests and respective tolerances to be computed in a simulated water phantom in the planning module of the TPS. For these tests, no additional measurements are required. Test 5.1 is intended to confirm that the doses calculated to the water phantom in the modeling and planning modules are identical to within the expected statistical uncertainty (considering noise and calculation grid size). This comparison should be performed using a large field for which commissioning data were acquired. Dose at several points should be confirmed. In Test 5.2, a beam equivalent to the beam calibration geometry should be planned in the TPS to ensure that the dose per MU matches the measured value at the calibration depth (e.g., 10 cm). Test 5.3 confirms that in the planning module, dose calculated to the water phantom matches a subset of input commissioning measurements. For this test, parameters such as PDD, off axis factors, and output factors should be compared for a large and a small field collected during commissioning.

Table 4 describes validation tests that use field configurations different from those used for modeling. For example, some TPSs require model data be taken at 100 cm SSD; therefore, validation tests should be performed at other SSDs. Field shaping should be accomplished using the MLC with jaws placed at clinically relevant positions for tests 5.4–5.6. The MLC and/or jaws may be used for tests 5.7–5.9. Sample setups for the tests are described in the IAEA TRS Report 430.^(7)^ Tests 5.4 through 5.8 should be done for each unique beam. Physical wedged beams are considered unique. Test 5.8 tests the TPS systems ability to calculate dose for beams that are oblique to the surface. If done in a water tank this should be done at the largest angle possible. This can be tested at larger angles if done in solid phantom. Test 5.9 is specific to nonphysical wedges. For all tests, measurements in the high‐dose region, penumbra, and low‐dose tail regions should be compared to calculated values at various depths (including slightly beyond $d_{\text{max}}$, midrange/10–15 cm, and deep/25–30 cm) and off‐axis positions. Table 5 summarizes the evaluation methods and tolerances for basic photon tests in Table 4. For an inverse planning only TPS (e.g., tomotherapy), the basic photon tests can be performed by creating simple targets and optimizing a plan for each case (e.g., small, large, on/off‐axis, variable SSD) or by using computed data for static fields provided by the vendor.

**Table 3:** TPS model comparison tests and tolerances

*Test*	*Comparison*	*Description*	*Tolerance*
5.1	Dose distributions in planning module vs. modeling (physics) module	Comparison of dose distribution for large ($>30\times 30\;{\text{cm}}^{2}$) field.	Identical^a^
5.2	Dose in test plan vs. clinical calibration condition^b^	Reference calibration condition check	0.5%
5.3	Dose distribution calculated in planning system vs. commissioning data	PDD and off axis output factors for a large and a small field size	2%

a Identical to within the expected statistical uncertainty (considering noise and calculation grid size).

b TPS absolute dose at reference point.

**Table 4:** Basic photon beam validation tests summary^a^

*Test*	*Description*	*Sample tests from literature* ^(7)^
5.4	Small MLC‐shaped field (non SRS)	Photon Test 1
5.5	Large MLC‐shaped field with extensive blocking (e.g., mantle)	Photon Test 3
5.6	Off‐axis MLC shaped field, with maximum allowed leaf over travel	Photon Test 2
5.7	Asymmetric field at minimal anticipated SSD	Photon Test 6
5.8	$10\times 10\;{\text{cm}}^{2}$ field at oblique incidence (at least 20°)	Photon Test 10
5.9	Large (>15 cm) field for each nonphysical wedge angle^b^	–

a For all tests, measurements in the high‐dose region, penumbra, and low‐dose tail regions should be compared to calculated values at various depths (including slightly beyond dmax, midrange/10–15 cm, and deep/25–30 cm). SSDs, other than those used at commissioning and that reflect the clinically expected range, should be used. The MLC should be used for tests 5.4–5.6. The MLC or jaws may be used for tests 5.7–5.9.

b Tests 5.4–5.8 are intended for each open and (hard) wedged field. Nonphysical wedges are considered an extension of the corresponding open field in terms of spectra and only require the addition of Test 5.9.

**Table 5:** Basic TPS photon beam evaluation methods and tolerances

*Region*	*Evaluation Method*	*Tolerance* ^a^ *(consistent with IROC Houston)*
High dose	Relative dose with one parameter change from reference conditions	2%
	Relative dose with multiple parameter changes^b^	5%
Penumbra	Distance to agreement	3 mm
Low‐dose tail	Up to 5 cm from field edge	3% of maximum field dose

a Tolerances are relative to local dose unless otherwise noted.

b For example, off‐axis with physical wedge.

As discussed in the introduction, TPS modeling is an iterative process that includes compromises in accuracy over the range of clinical scenarios that could be encountered. In the spirit of minimum practice guidelines, these basic photon tolerance values, especially in the situation with multiple parameter changes (e.g., an off‐axis measurement in the presence of a wedge) are the ‘worst case scenarios.’ Some aspects of the tests in Table 4 may show excellent agreement, while others may show poorer agreement. This report recommends that the results of the validation tests should meet criteria consistent with those of IROC Houston. These tolerances are summarized in the recommendations below and in Table 5

### B. Recommendations

- The reference condition dose per MU should match within 0.5% (Test 5.2).
- The relative dose distributions calculated by the TPS should match measured values in the high‐dose regions at different depths and off‐axis positions to within 2% for fields with one parameter changed from the reference conditions.
- For fields with multiple parameter changes (e.g., an off‐axis measurement in the presence of a wedge), disagreement up to 5% is allowed. It is further noted that the majority of the validation experiments should display significantly better agreement than 5%, and if a large number of the results are near this tolerance then additional model improvement should be investigated.
- The penumbra should match with a 3 mm distance to agreement.
- The low‐dose profile tails, up to 5 cm from field edge, computed by the TPS should agree with measurement to within 3% of the maximum field dose.

Users should always strive for the best possible agreement between modeled and measured results. The QMP must understand the limitations of the dose calculation algorithm in measurement conditions such as the buildup region, oblique incidence, and penumbra. While it may be deemed clinically acceptable for the TPS to disagree with the delivered dose by more than the above criteria, these cases must be understood, clinically justified, and documented. It is important to reiterate that, if the model barely passes the basic photon recommendations on a machine that will also be used for IMRT/VMAT, the dosimetric agreement for IMRT/VMAT plans will be poor. It is also recognized that additional modeling for the IMRT/VMAT may affect the parameter results of the basic photon beam modeling, specifically the penumbra and tails. It is also important to recognize this as a workflow issue. Once IMRT modeling is completed, the basic beam modeling will need to be rechecked. Consequently, the physicist may want to conduct IMRT/VMAT modeling before basic photon modeling is finalized.

## 6. PHOTON BEAMS: HETEROGENEITY CORRECTION VALIDATION

The commissioning of heterogeneity corrections requires the accurate commissioning of the beam itself and accurate characterization of the patient data. For dose calculation in heterogeneous media (e.g., the thorax), modern and advanced algorithms such as C/S, CC, GBBS, or MC are required, and PB and correction based algorithms are unacceptable. Many studies detail the accuracy of these algorithms.^26^, ^27^, ^28^, ^29^, ^30^, ^31^ The QMP must understand not only the implementation of their heterogeneity corrections, but also their limitations, particularly in the context of known dose discrepancies, which should be distinguished from incorrect implementation/commissioning of the TPS. Particular care should be taken when evaluating calculated dose 1) within low‐density tissue, 2) near the interface of heterogeneous tissues, and 3) beyond low/high density tissue. A detailed overview of many types of heterogeneity corrections and tests can be found in the AAPM Report 85^(32)^ and IAEA TRS Report 430.^(7)^

Monte Carlo and GBBS algorithms directly calculate dose to the material within the voxel (“dose to medium”). This can be converted to “dose to water” through application of stopping power ratios, with the goal of reproducing conventional (e.g., C/S) TPS doses.^(33)^ However, this stopping power‐based conversion has actually been found to decrease dosimetric agreement with conventional TPS doses in most cases,^34^, ^35^ leading to “dose to medium” being recommended.^(34)^ Nevertheless, further study is warranted and vendors are currently encouraged to calculate both dose to medium and dose to water; the QMP must be aware of which dose is being reported.

### A. Validation tests

The recommended minimum validation of the heterogeneity calculations includes confirmation of the CT‐density table and basic measurement tests of TPS calculations beyond lung heterogeneities. The implementation of each CT‐density table must be verified in the TPS. For scanners that will be considered equivalent, the QMP should verify calculations with data from each scanner.

Table 6 summarizes the validation testing for TPS dose calculation in heterogeneous media. Test 6.1 is a simple verification that the TPS‐reported densities match the actual densities of the phantom.^(7)^ Test 6.2 verifies dose beyond low‐density (lung) material. Any heterogeneous phantom available can be used for these measurements. A reasonable slab phantom setup is found in Carrasco et al.^(28)^ It consists of a 5 cm slab of water‐equivalent plastic stacked upon a 13 cm slab of lung‐equivalent material, upon a 10 cm slab of water‐equivalent plastic. For lung‐equivalent material, any type of low‐density material, such as low‐density wood (approximately $0.3\;g/{\text{cm}}^{3}$), can be used, as long as the thickness is sufficient to result in a dose correction greater than 10% compared to a homogeneous phantom. For Test 6.2, the ratio of the dose values above and below the heterogeneous medium along the central axis must be measured and compared with TPS calculated ratio according to the following:

- Measurements should be made outside of the buildup/builddown regions.^(26)^ This simple test allows for the direct study of the calculation accuracy through the heterogeneity.
- The recommended field size is $5\times 5\;{\text{cm}}^{2}$ because discrepancies due to low‐density material tend to be exacerbated at smaller field sizes.
- Further tests deemed appropriate by the QMP to challenge the accuracy of the particular calculation algorithm being employed should be used to bring a better understanding of the limitations of dose calculation in the vicinity of heterogeneities.

**Table 6:** Heterogeneous TPS photon beam validation tests

*Test*	*Objective*	*Description*	*Tolerances* ^a^	*Reference*
6.1	Validate planning system reported electron (or mass) densities against known values	CT‐density calibration for air, lung, water, dense bone, and possibly additional tissue types	–	TG 65,^(26)^ IAEA TRS‐430^(7)^
6.2	Heterogeneity correction distal to lung tissue	$5\times 5\;{\text{cm}}^{2}$, measure and calculate dose ratio above and below heterogeneity, outside of the buildup region	3%	IAEA TRS‐430,^(7)^ Carrasco et al.^(28)^

a Tolerances are relative to local dose unless otherwise noted.

### B. Recommendations

- To produce acceptable dosimetric accuracy in highly heterogeneous media (particularly in lung), an algorithm comparable to C/S, CC, MC, or GBBS‐based dose calculation algorithm must be used.
- The QMP should understand the implementation and limitations of the heterogeneity corrections used in the chosen algorithm.
- The CT to density curve, as discussed in Section 3B, should be used to accurately construct a CT‐density table within the TPS and should be verified (Test 6.1).
- The impact of low‐density heterogeneities on central axis dose should be quantitatively verified with a recommended 3% dose agreement beyond lung‐equivalent material (Test 6.2).

## 7. PHOTON BEAMS: IMRT/VMAT DOSE VALIDATION

This section describes the final dosimetric commissioning step for photons — comparison of the individual beams and/or composite measurements of IMRT/VMAT/helical delivery plans with TPS calculations. IMRT/VMAT dose validation has the least amount of consensus amongst medical physicists and is controversial. Despite widespread IMRT utilization, accurate dosimetric commissioning of an IMRT system remains a challenge. In the most recent report from IROC Houston,^(36)^ only 82% of the institutions passed the credentialing end‐to‐end test with the anthropomorphic head and neck phantom. That test used rather lenient dose‐ratio and distance‐to‐agreement (DTA) criteria of 7% and 4 mm, respectively. Only 69% percent of the irradiations passed a narrowed TLD dose‐error criterion of 5%. A substantial amount of the failures were traced to the fundamentals of the TPS commissioning. As such, the approach and acceptance criteria used for dosimetric commissioning of IMRT/VMAT are of paramount importance.

### A. Validation tests

In Table 7, there are five types of validation tests recommended for IMRT/VMAT delivery modalities. Once the initial tests plans are developed with the most frequent energy (often 6 MV), the plans can be recalculated for the remaining energies, if applicable. If multiple delivery techniques are available for the same accelerator (e.g., segmental IMRT, dynamic IMRT, VMAT, tomotherapy), each one must be validated separately.

Test 7.1 is a verification of small‐field PDD. As mentioned in the data acquisition section, the TPS may not require small‐field depth doses for beam modeling. The verification is important since extrapolated data will effectively be used for planning. This can help in understanding the limits of the TPS. If the TPS is provided with a predefined beam model, then the QMP should request reference data for these measurements from the vendor.

Test 7.2 is verification of small MLC fields not explicitly used in beam modeling. This test differs from the small‐field MLC basic test (Test 5.4), which represents a field that could be clinically used on its own. Since the gap between opposed leaves can be 1 cm or less in IMRT/VMAT, it is imperative to measure the output of small MLC shaped fields, including IMRT‐type fields where the jaws are substantially more open than the MLC “opening”. The QMP should measure output factors down to a field size of $2\times 2\;{\text{cm}}^{2}$ (and preferably smaller) for a clinically relevant depth, then compare the measured results to the treatment planning system calculations.^(22)^ The jaws should be positioned to reflect their state during the IMRT/VMAT field delivery.

**Table 7:** VMAT/IMRT test summary

*Test*	*Objective*	*Description (example)*	*Detector*	*Ref*
7.1	Verify small field PDD	$\leq 2\times 2\;{\text{cm}}^{2}$ MLC shaped field, with PDD acquired at a clinically relevant SSD	Diode or plastic scintillator	Yunice et al^.(16)^
7.2	Verify output for small MLC‐defined fields	Use small square and rectangular MLC‐defined segments, measuring output at a clinically relevant depth for each^a^	Diode, plastic scintillator, minichamber or microion chamber	Cadman et al.^(58)^
7.3	TG‐119 tests	Plan, measure, and compare planning and QA results to the TG119 report for both the Head and Neck and C‐shape cases	Ion chamber, film and/or array	TG‐119 (Ezzell et al.^(37)^)
7.4	Clinical tests	Choose at least 2 relevant clinical cases; plan, measure, and perform an in‐depth analysis of the results	Ion chamber, film and/or array	Nelms et al.^(42)^
7.5	External review	Simulate, plan, and treat an anthropomorphic phantom with embedded dosimeters.	Various options exist^b^	Kry et al.^(39)^

a A bar pattern scanned with a diode can be used to obtain additional absolute dose profile comparison in the direction perpendicular to MLC movement

b If IROC Houston service is used, they typically employ TLDs and radiochromic film. Certain commercial phantoms can accommodate ion chambers for point dose measurements

The remaining test plan strategy follows the progression from simple to more complex clinical implementation. Test 7.3 recommends two plans from the TG‐119 test suite^(37)^ as a starting point: Mock Head and Neck and C‐shape. Test 7.4 recommends using at least two image sets for optimization and delivery verification that are representative of the intended clinical cases to be treated. Each modality (e.g., IMRT, VMAT, tomotherapy) and energy must be separately validated. Users can use their own cases or download sample datasets and objectives from http://www.aapm.org/pubs/MPPG/TPS/. Test plans should use the same dose grid resolution (and angular control point resolution for VMAT) that will be used clinically. Tests 7.3 and 7.4 should also be used to test the patient‐specific QA process. The plans should be delivered to a phantom with appropriate dosimeters that will enable the user to compare planned and delivered dose distributions.^(38)^

Test 7.5 is a complete end‐to‐end test that involves scanning an anthropomorphic phantom, planning, delivery, and sending dosimeters out for external review. At least one end‐to‐end test must be performed for each commissioned modality. A head and neck plan, such as the IROC Houston credentialing test,^(36)^ is encouraged, as complicated test plans are more likely to demonstrate possible commissioning deficiencies. In addition, if the facility is planning to employ IMRT/VMAT in the thoracic region, a second end‐to‐end test with a heterogeneous thoracic phantom should be performed.^(39)^ Even with modern model‐based dose calculation algorithms, systematic differences between calculated and measured doses in lung have been noted^39^, ^40^ and can be worsened by user‐configurable parameters. It is worth noting that participation in clinical trials is no longer required to obtain such evaluation on a fee‐for‐service basis. If a formal third‐party mail‐in dosimetry evaluation is not possible, the results of the end‐to‐end tests should, at minimum, be peer‐reviewed by an independent QMP.

### B. Recommendations

- The range of optimization parameters (e.g., amount of modulation, minimum field size) and types of plans tested during commissioning should be clearly documented and representative of clinical practice. As clinical practices change, it may be necessary to conduct additional validation to accommodate new planning techniques.
- In general, more time should be devoted to fine tuning the model for the highly modulated plans. Simple plans are typically much less influenced by the often‐changed parameters, particularly the parameters related to the MLC model.^(41)^
- The recommended evaluation criteria provided in Table 8 refer to composite dose distributions.^(42)^
- The average difference between ion chamber and TPS doses in the low‐gradient target region should not exceed 2%, with less than 1.5% preferred.^(37)^ In regions of organs at risk (OAR), TG‐119 findings (agreement within 3% of prescription dose) are generally appropriate. The locally normalized dose difference should also be evaluated for areas and patterns of disagreement.
- Planar or volumetric measurements (film or an electronic array with appropriate effective resolution) should be evaluated with 2%/2 mm gamma analysis to emphasize areas of disagreement. Application of a 2%/2 mm gamma criterion can result in the discovery of easily correctable problems with IMRT commissioning that may be hidden in the higher (and ubiquitous) 3%/3 mm passing rates.^(40)^
- There is even less guidance on the optimal criteria for end‐to‐end anthropomorphic test accuracy. This report recommends that the institution strive for a 5% agreement, which is consistent with IROC Houston criteria.
- Additional verification testing may be required for treatment scenarios specific to a given delivery modality or linear accelerator. For example, if the MLC carriage does not move during delivery it may be necessary to split fields, which can produce repetitive MLC leaf junction patterns that are sensitive to MLC modeling parameters. In addition, the treatment couch should be accounted for during IMRT/VMAT treatments that contain beams delivered through the couch.^43^, ^44^

**Table 8:** VMAT/IMRT evaluation methods and tolerances

*Measurement Method*	*Region*	*Tolerance*
Ion Chamber	Low‐gradient target region OAR region	2% of prescribed dose
		3% of prescribed dose
Planar/Volumetric Array	All regions	2%/2 mm ^a^, no pass rate tolerance, but areas that do not pass need to be investigated
End‐to‐End	Low‐gradient target region	5% of prescribed dose

a Application of a 2%/2 mm gamma criterion can result in the discovery of easily correctable problems with IMRT commissioning that may be hidden in the higher (and ubiquitous) 3%/3 mm passing rates.^(39)^

## 8. ELECTRON BEAM VALIDATION

The AAPM TG‐25 Report^(45)^ and its supplement AAPM TG‐70 Report^(46)^ provide extensive detail on electron dosimetry. This current report is based on the AAPM TG‐70 Report recommendation that “... treatment planning for electron beams should be CT data based, employ 3‐D heterogeneity corrections and, at a minimum, use [Pencil Beam] PB‐based algorithms.” The following validation tests are recommended for routine electron therapy generated with image‐based electron planning systems employing 3D dose calculation algorithms (PB and MC). The accuracy and use of PB algorithms are well documented.^47^, ^48^, ^49^, ^50^ Monte Carlo algorithms are becoming a common practice in commercially available systems.^51^, ^52^, ^53^, ^54^

As with photons, electron commissioning data should be collected in air and water as specified by the vendor.^(55)^ Once the beam has been optimally modeled in the TPS, additional validation tests should be conducted to test the system's ability to calculate isodose distributions in nonstandard setups (e.g., patient specific cutouts, oblique incidence, extended SSD and heterogeneous media). Much of the data for these validation tests can be obtained in water at the same time as the standard field data (used for modeling) is acquired. Other suitable phantom/detector combinations, such as array detectors, may also be used for validation measurements with consideration of the limitations of each device for electron dosimetry.^(56)^ The QMP should balance the advantages and disadvantages of different measurement devices when performing the following validation tests. Water tank profiles yield the most accurate absolute dose comparison, while array detectors can test multiple points within the distribution and provide efficient comparison to calculations.

### A. Validation tests

Three tests are summarized in Table 9 for validation of the TPS electron dose calculation algorithm. Test 8.1 compares the calculated isodose distribution for a custom cutout to a measured distribution at a standard and extended SSD. The cutout field size must be large enough to provide lateral scatter equilibrium.^(46)^ This will test both the system's ability to calculate dose with a custom cutout and verify that the virtual/effective SSD calculation is being applied correctly. This test should be performed for all energies.

Test 8.2 compares measured to calculated isodose distributions for an obliquely incident beam. This will test the impact of central axis tilt on depth dose and penumbra. This test should be performed in a homogeneous medium at the nominal clinical SSD. This test should be performed for all energies.

Test 8.3 tests the electron dose calculation algorithm in the presence of heterogeneities. A calculation setup similar to the photon heterogeneity test described in Section 6 can be used. At a minimum, this test should be performed for one energy at a suitable depth. Dose distributions from the TPS should be qualitatively compared to expected values.

**Table 9:** Basic TPS validation tests for electron beams and minimum tolerance values

*Test*	*Objective*	*Description*	*Tolerance*
8.1	Basic model verification with shaped fields	Custom cutouts at standard and extended SSDs	3%/3 mm
8.2	Surface irregularities obliquity	Oblique incidence using reference cone and nominal clinical SSD	5%
8.3	Inhomogeneity test	Reference cone and nominal clinical SSD	7%

### B. Recommendations

- Plot PDD and output factors for all cones (with standard cutout sizes) for each energy to confirm the correct qualitative behavior as a function of field size and energy.
- For normal incidence (Test 8.1), measured and calculated isodose distributions should be within 3% agreement in the high‐dose region/low‐dose gradient and 3 mm DTA for PDDs along the central axis (excluding the buildup region). Note that percentages are of the central ray normalization dose.
- For oblique incidence (Test 8.2), measured and calculated isodose distributions should agree within 5% in the high‐dose, low‐dose gradient region.^(57^)
- Heterogeneity corrected manual dose calculations (Test 8.3) for the institution's heterogeneous phantom should be compared with CT‐based calculations.^(7^) This comparison is generally qualitative, but should not exceed more than 7%.
- Clinically used nonroutine electron setups (e.g., abutting electron/electron fields, electron/photon fields, and small fields that results in a loss of lateral electron equilibrium) will require additional dosimetric verification to understand the limits of the electron dose model.

## 9. ESTABLISHING ROUTINE QA

Once commissioning has been completed, the QMP should establish a routine QA program to ensure that 1) the TPS has not been unintentionally modified, and 2) dose calculation is consistent following TPS upgrades. Unintentional modifications can be identified through the use of file integrity checksums.^(6)^, ^‡^ TPS vendors should provide methods for performing checksums on their respective systems. Dose calculation consistency can be performed by recalculating a subset of the tests defined in Sections 5‐8 of this report. This report recommends these tests be conducted annually or following TPS system upgrades.

Routine TPS QA complements machine QA, which validates the integrity of the linac output, MLC position, and other delivery parameters. Measurements are not required for TPS QA. Rather, each sample plan should be recalculated and compared to the baseline obtained during commissioning. Additional TPS checks, such as DVH calculation, effective depth calculation, and CT number consistency, can be performed using the same datasets.

### A. Recommendations

- Reference plans should be selected at the time of commissioning and then recalculated for routine QA comparison.
- For photons, representative plans for each configured beam should be chosen from Table 4 for static and wedge beams and Table 7 for IMRT/VMAT.
- For electrons, sample plans should be calculated for each energy using a heterogeneous dataset with reasonable surface curvature. It is also recommended to include extended distance and bolus verification in the sample plans.
- Optionally, an additional thorax dataset with contours and suggested static beam parameters is included with the downloadable IMRT/VMAT sample datasets (http://www.aapm.org/pubs/tg244/). The curvature and inhomogeneity conditions of this dataset are applicable for TPS dose algorithm testing of wedged fields, dynamic arc, and/or electron plans.
- All routine QA recalculations should agree with the reference dose calculation to within 1%/1 mm. A partial or complete recommissioning (including validation) may be required if more significant deviations are observed.

## 10. SUMMARY

The guidelines and recommendations provided in this report should aid the QMP in beam data acquisition, modeling, validation, and establishment of baseline routine QA datasets for their TPS. The QMP can substitute, alter, or add to the recommended test suite as needed, as long as the change is made in the spirit of fulfilling or exceeding the minimum practice guidelines described within each section and justification is appropriately documented. As with all MPPG, this current report summarizes only a minimum scope of work that is necessary in the clinical setting.

Through completion of these guidelines, the QMP should also have improved his/her understanding of the strengths and limitations of the TPS beam model and dose calculation algorithms. Many TPS vendors provide guidance on expected levels of accuracy under different scenarios. The QMP should understand why these limitations exist, and use them as a guide when evaluating the accuracy of their beam model. Knowledge of these limitations can also help define under what clinical applications the TPS is appropriate, and if new applications will require additional fine‐tuning or adjustment to the beam model.

Through the entire commissioning process, it is imperative to maintain clear and thorough documentation of the tests performed, equipment used, results, and findings. This documentation must be compiled into a final commissioning report by the QMP, and appended with future TPS modification or recommissioning documentation. Appendix A is an optional Dose Algorithm Commissioning Inventory that can assist the QMP in determining whether the major tasks outlined in this document have been accomplished. It can also serve as a guideline for documentation.

Finally, the importance of peer review should be reiterated. Peer review of the TPS model parameters, agreement to measured data, and validation procedure/results is highly recommended. This should include independent dose calculations of basic dosimetry parameters (determined by another physicist) compared with independent measurements (also made by the other physicist).

## ACKNOWLEDGMENTS

This guideline was developed by the Medical Physics Practice Guideline Task Group‐244 of the Professional Council of the AAPM.

MPPG 5 Members:

Jennifer Smilowitz, Chair, PhD

Indra J. Das, PhD, FAAPM, FACMP

Vladimir Feygelman, PhD

Benedick A. Fraass, PhD, FAAPM, FASTRO, FACR

Mark W. Geurts, MS

Stephen F. Kry, PhD

Ingrid R. Marshall, PhD

Dimitris N. Mihailidis, PhD, FAAPM, FACMP

Zoubir Ouhib, MS

Timothy Ritter, PhD

Michael G. Snyder, PhD, FCCMP

Lynne A. Fairobent, AAPM Staff

AAPM Subcommittee on Practice Guidelines — AAPM Committee responsible for sponsoring the draft through the process:

Russell Tarver, MS, Chair

Maria F. Chan, PhD, FAAPM, Vice‐Chair Therapy

Jessica B. Clements, MS

Jonas D. Fontenot, PhD

Luis E. Fong de los Santos, PhD

Arthur J. Olch, PhD, FAAPM

Joann I. Prisciandaro, PhD, FAAPM

J. Anthony Seibert, PhD, FAAPM, FACR S Jeff Shepard, MS, FAAPM, Vice‐Chair Imaging

Jennifer Smilowitz, PhD

James J. VanDamme, MS

Gerald A. White Jr., MS, FAAPM

Lynne A. Fairobent, AAPM Staff

## Purpose

This optional commissioning checklist can assist the QMP in determining whether the major MPPG tasks have been accomplished and appropriately documented in the commissioning report. This inventory should be used only after the report has been read in its entirety.

## Definition

A beam is typically distinguished as a unique energy and/or accelerator head model configuration. Note: Each physical wedge or universal wedge is a unique beam because of its unique energy fluence spectrum and should be separately validated.

**Table:**

*MPPG Section*	*MPPG Item for Each Beam Model*	*Commissioning Report Pages*
1	QMP understands algorithms and has received proper training.	
3	Manufacturer's guidance for data acquisition was consulted and followed.	
3.B	Appropriate CT calibration data acquired.	
3.D	Review of raw data (compare with published data, check for error, confirm import into TPS).	
4	Beam modeling process completed according to manufacturer's instructions.	
4	Beam models evaluated qualitatively and quantitatively using metrics within the modeling software.	
5	Basic photon beam validation: Tests 5.1–5.8 (5.9 for nonphysical wedge).	
6	Heterogeneity correction validation for photon beams: Tests 6.1–6.2	
7	IMRT/VMAT validation: Tests 7.1–7.4	
7	IMRT/VMAT End‐to‐End test with external review: Test 7.5	
7	Understand and document limitations of IMRT/VMAT modeling and dose algorithms.	
8	Electron validation: Tests 8.1–8.3	
9	Baseline QA plan(s) (for model constancy) identified for each configured beam and routine QA established.	
